# Reticulophagy and viral infection

**DOI:** 10.1080/15548627.2024.2414424

**Published:** 2024-10-12

**Authors:** Alexa Wilson, Craig McCormick

**Affiliations:** Department of Microbiology & Immunology, Dalhousie University, Halifax, Nova Scotia, Canada

**Keywords:** Autophagy, endoplasmic reticulum, reticulophagy, unfolded protein response, virus

## Abstract

All viruses are obligate intracellular parasites that use host machinery to synthesize viral proteins. In infected eukaryotes, viral secreted and transmembrane proteins are synthesized at the endoplasmic reticulum (ER). Many viruses refashion ER membranes into bespoke factories where viral products accumulate while evading host pattern recognition receptors. ER processes are tightly regulated to maintain cellular homeostasis, so viruses must either conform to ER regulatory mechanisms or subvert them to ensure efficient viral replication. Reticulophagy is a catabolic process that directs lysosomal degradation of ER components. There is accumulating evidence that reticulophagy serves as a form of antiviral defense; we call this defense “xERophagy” to acknowledge its relationship to xenophagy, the catabolic degradation of microorganisms by macroautophagy/autophagy. In turn, viruses can subvert reticulophagy to suppress host antiviral responses and support efficient viral replication. Here, we review the evidence for functional interplay between viruses and the host reticulophagy machinery.

**Abbreviations**: AMFR: autocrine motility factor receptor; ARF4: ADP-ribosylation factor 4; ARL6IP1: ADP-ribosylation factor-like 6 interacting protein 1; ATL3: atlastin GTPase 3; ATF4: activating transcription factor 4; ATF6: activating transcription factor 6; BPIFB3: BPI fold containing family B, member 3; CALCOCO1: calcium binding and coiled coil domain 1; CAMK2B: calcium/calmodulin-dependent protein kinase II, beta; CANX: calnexin; CDV: canine distemper virus; CCPG1: cell cycle progression 1; CDK5RAP3/C53: CDK5 regulatory subunit associated protein 3; CIR: cargo-interacting region; CoV: coronavirus; CSNK2/CK2: casein kinase 2; CVB3: coxsackievirus B3; DAPK1: death associated protein kinase 1; DENV: dengue virus; DMV: double-membrane vesicles; EBOV: Ebola virus; EBV: Epstein-Barr Virus; EIF2AK3/PERK: eukaryotic translation initiation factor 2 alpha kinase 3; EMCV: encephalomyocarditis virus; EMV: extracellular microvesicle; ER: endoplasmic reticulum; ERAD: ER-associated degradation; ERN1/IRE1: endoplasmic reticulum to nucleus signalling 1; EV: extracellular vesicle; EV71: enterovirus 71; FIR: RB1CC1/FIP200-interacting region; FMDV: foot-and-mouth disease virus; HCMV: human cytomegalovirus; HCV: hepatitis C virus; HMGB1: high mobility group box 1; HSPA5/BiP: heat shock protein 5; IFN: interferon; IFNG/IFN-γ: interferon gamma; KSHV: Kaposi’s sarcoma-associated herpesvirus; LIR: MAP1LC3/LC3-interacting region; LNP: lunapark, ER junction formation factor; MAP1LC3: microtubule-associated protein 1 light chain 3; MAP3K5/ASK1: mitogen-activated protein kinase kinase kinase 5; MAPK/JNK: mitogen-activated protein kinase; MeV: measles virus; MHV: murine hepatitis virus; NS: non-structural; PDIA3: protein disulfide isomerase associated 3; PRR: pattern recognition receptor; PRRSV: porcine reproductive and respiratory syndrome virus; RB1CC1/FIP200: RB1-inducible coiled-coil 1; RETREG1/FAM134B: reticulophagy regulator 1; RHD: reticulon homology domain; RTN3: reticulon 3; RTN3L: reticulon 3 long; sAIMs: shuffled Atg8-interacting motifs; SARS-CoV: severe acute respiratory syndrome coronavirus; SINV: Sindbis virus; STING1: stimulator of interferon response cGAMP interactor 1; SVV: Seneca Valley virus; SV40: simian virus 40; TEX264: testis expressed gene 264 ER-phagy receptor; TFEB: transcription factor EB; TRAF2: TNF receptor-associated factor 2; UIM: ubiquitin-interacting motif; UFM1: ubiquitin-fold modifier 1; UPR: unfolded protein response; VAPA: vesicle-associated membrane protein, associated protein A; VAPB: vesicle-associated membrane protein, associated protein B and C; VZV: varicella zoster virus; WNV: West Nile virus; XBP1: X-box binding protein 1; XBP1s: XBP1 spliced; xERophagy: xenophagy involving reticulophagy; ZIKV: Zika virus

## Introduction

The endoplasmic reticulum (ER) is a hub for cellular anabolic activities including synthesis of proteins, lipids, and steroids. It is divided into sub-compartments with distinct properties that support these activities [1–3]. ER sheets have a flat appearance that resembles stacked pancakes with curved ends, studded with ribosomes that translate secreted and transmembrane proteins [4]. Radiating from ER sheets are ER tubule networks that serve as sites for lipid synthesis and interactions with other organelles. This dynamic organelle senses and responds to cell stress and adapts to cellular biosynthetic demands; it requires substantial resources to meet these demands and maintain protein homeostasis, also known as “proteostasis”. Exceeding ER protein folding capacity causes the accumulation of nascent unfolded proteins in the ER lumen, which activates three ER-localized sensor proteins, EIF2AK3/PERK (eukaryotic translation initiation factor 2 alpha kinase 3), ERN1/IRE1 (endoplasmic reticulum to nucleus signaling 1) and ATF6 (activating transcription factor 6) [5,6]. Each ER stress sensor converts activation signals into the synthesis of transcription factors that relocate to the cell nucleus and transactivate genes involved in increasing ER protein folding and processing capacity to satisfy demand; this is known as the unfolded protein response (UPR). In addition to these transcriptional responses, the UPR also reduces ER load by EIF2AK3-dependent arrest of cap-dependent translation initiation, and by increasing ER-associated catabolic activities [7,8]. Chief among these is “reticulophagy”, a catabolic process whereby ER cargo is diverted to the lysosome for degradation. Upon ER stress resolution, reticulophagy resets ER capacity to normal levels by disposing of surplus ER membranes, enzymes, and chaperones. Thus, reticulophagy is an essential player in maintaining ER homeostasis.

### Reticulophagy at-a-glance

Autophagy is a catabolic process involving autophagosomes and lysosomes that work together to degrade cellular contents to liberate macromolecules and energy to promote cellular homeostasis. The phenomenon that would eventually be known as reticulophagy was first documented in 1973 by Bolender and Weibel, who observed accumulation of chemically-expanded ER membranes in vacuoles in rat hepatocytes [9]. This initial observation was confirmed decades later by Ohsumi and Walter, who observed accumulation of ER membranes in the yeast vacuole in response to rapamycin-induced starvation stress [10] and ER stress [11], respectively. Walter coined the term “ER-phagy” (now referred to as “reticulophagy”) to describe this new form of organelle-specific autophagy to complement mitophagy (autophagic degradation of mitochondria) and pexophagy (autophagic degradation of peroxisomes). Subsequent studies established at least two distinct mechanisms: i) macroreticulophagy, whereby bulk ER is engulfed by autophagosome membranes that subsequently fuse with lysosomes to generate autolysosomes, or ii) microreticulophagy, whereby smaller ER fragments are directly engulfed by lysosomes. A hallmark of macroreticulophagy, hereafter reticulophagy, is the involvement of ER localized cargo receptors that interact with autophagosome membranes to target portions of the ER for autophagic degradation. Reticulophagy can be upregulated to meet increased demands to maintain ER proteostasis in adverse conditions, including nutrient scarcity [12–14] or UPR activation [15]. Recently, reticulophagy has been implicated in host antiviral responses, an idea supported by accumulating evidence for viral mechanisms of reticulophagy subversion [16–22]. This viral control of reticulophagy is primarily focused on reticulophagy receptors that are spatially organized within ER domains and specifically recruit ER-fragments and/or ER-resident proteins for degradation in the autolysosome. The following section will provide a detailed overview of each of the known reticulophagy receptors.

## Transmembrane, reticulon homology domain-containing reticulophagy receptors

### RETREG1/FAM134B

**Figure 1.**
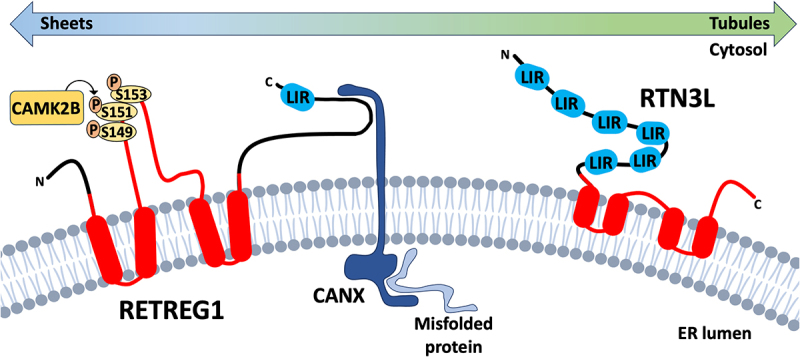
Reticulon homology domain-containing reticulophagy receptors RETREG1 and RTN3L. Reticulon homology domain (rhd)-containing reticulophagy receptors are comprised of transmembrane or membrane-associated RHD domains (in red) connected to domains involved in substrate recognition and LC3 binding. RETREG1 is primarily found in ER sheets where it captures misfolded substrate proteins bound to the ER transmembrane chaperone CANX. ER stress triggers post-translational modifications of the RETREG1 RHD, including CAMK2B-mediated phosphorylation, that contribute to RETREG1 oligomerization and er-scission, while LC3 binding recruits phagophores to initiate local degradation of ER membranes and cargo. RTN3L is primarily associated with the cytoplasmic leaflet of ER tubules, and relatively little is known about substrate recognition mechanisms. Six LIRs on the cytoplasmic amino-terminal domain direct binding to Atg8-like proteins and phagophore recruitment.

The discovery of the first mammalian reticulophagy receptor, RETREG1/FAM134B (reticulophagy regulator 1) was a significant breakthrough that energized the reticulophagy field [23]. RETREG1 is a multi-pass transmembrane protein primarily found in ER sheets that features a reticulon homology domain (RHD) that allows it to accumulate in areas of high local membrane curvature where it drives membrane fragmentation, a process known as ER scission [24] (Figure 1). Meanwhile, the carboxy-terminal LC3-interacting region (LIR) (Table 1) recruits nascent autophagosomes to initiate local degradation of ER membranes and cargo. RETREG1 plays an important role in regulating bulk ER volume and structure, as silencing RETREG1 expression yields a larger, extended ER morphology [23].

**Table 1.** Atg8-like protein interacting domains of reticulophagy receptors.

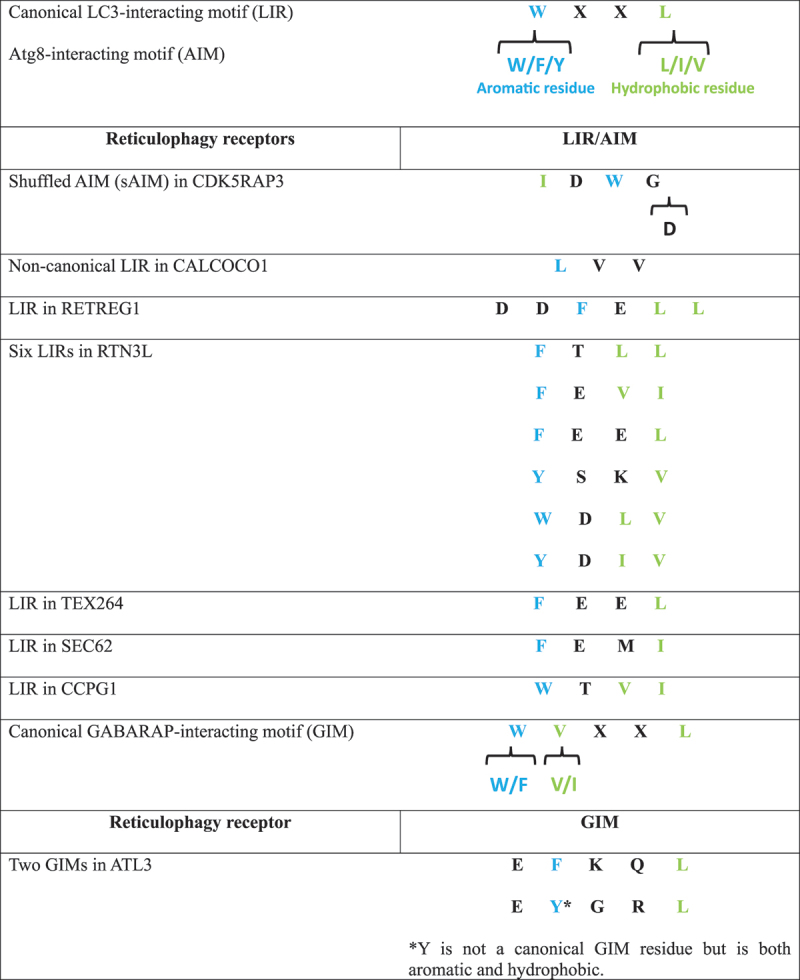

There is evidence for ER stress-dependent regulation of RETREG1 oligomerization and function. RETREG1 works in concert with the transmembrane ER chaperone CANX (calnexin) to target substrates for reticulophagy (Figure 1). Substrates identified to date include terminally misfolded procollagen [25], SNCA/α-synuclein [26], and NTRK2/tropomyosin receptor kinase B (neurotrophic tyrosine kinase, receptor, type 2) [27]. This reliance on CANX function provides a clear mechanistic link between RETREG1 and ER homeostasis mechanisms. ER stress also triggers RETREG1 acetylation by CREBBP (CREB binding protein) [28] and phosphorylation by CAMK2B (calcium/calmodulin-dependent protein kinase II, beta) [29]; these post-translational modifications contribute to RETREG1 oligomerization, ER scission and reticulophagy. BCL2 binding prevents BECN1 (beclin 1, autophagy related)-dependent autophagy [30]; interestingly, CAMK2B-mediated phosphorylation of BECN1 [31] releases it from BCL2 and induces autophagy, suggesting that CAMK2B regulates core autophagy machinery in addition to reticulophagy. Together, these studies contribute to the current model whereby RETREG1 identifies CANX-bound substrates, traffics to areas of high ER membrane curvature, drives ER scission via phosphorylation-dependent oligomer formation, and recruits nascent autophagosomes via interactions driven by the carboxy-terminal LIR.

Recently, RETREG2/FAM134A and RETREG3/FAM134C have also been linked to reticulophagy [32]. Like RETREG1, these multi-pass transmembrane proteins contain a RHD and a LIR (Table 1) and control ER size and morphology. However, RETREG2 and RETREG3 predominantly localize to ER tubules and induce ER scission at a slower rate than RETREG1. Proteomic analysis of cells deficient in RETREG1, RETREG2 or RETREG3 suggest that RETREG2 and RETREG3 share substrate specificity and regulate levels of the RETREG1 target misfolded procollagen [25].

There is a growing appreciation for the differential regulation of RETREG-family members by starvation stress. Several recent studies have investigated roles for nutrient-sensing pathways in the control of RETREG-mediated reticulophagy and provide evidence for phosphorylation of the RHD domain by CSNK2/CK2 (casein kinase 2) [33,34], which regulates ubiquitination and formation of high-density clusters of RETREG1 and RETREG3 on the ER membrane [34]. In this way, nutrient sensing pathways are linked to the RETREG receptor clustering that drives ER vesiculation and fragmentation during reticulophagy [24]. Mechanistically, the role for ubiquitination in this process is best understood for RETREG1. Many sites of RETREG1 ubiquitination have been mapped; ubiquitination of four lysine residues in the RHD promote *trans*-interactions between RHDs that support oligomerization, membrane-remodeling activity, and interactions with LC3/GABARAP-family proteins, thereby enhancing reticulophagy [34]. The E3 ubiquitin ligases that direct ubiquitination of RETREG1 remain incompletely characterized, but AMFR (autocrine motility factor receptor) ubiquitinates three lysine residues in the RHD; in turn, RETREG1 promotes the degradation of AMFR via reticulophagy [35]. Identification of additional E3 ubiquitin ligases that target residues in the RHD in RETREG1 and RTN3 (the other known reticulophagy receptor with an RHD) should significantly advance understanding of this aspect of reticulophagy receptor biology.

Another emerging theme in reticulophagy is the recruitment of auxiliary proteins to reticulophagy receptor complexes that contribute to ER-membrane curvature and scission. For example, RETREG1 binds an additional RHD-containing protein called ARL6IP1 (ADP-ribosylation factor-like 6 interacting protein 1) [36]. AMFR ubiquitinates ARL6IP1 in addition to RETREG1 in these multiprotein complexes to promote ER-remodeling during reticulophagy in ER sheets. By contrast, RETREG3 binds ER-localized reticulon RTN4 and the two proteins are enriched in ER tubules and sheet edges [37]. These findings suggest that RETREG-family members could be distributed in discrete ER subdomains to respond to unique stress stimuli, with the ER sheet-localized RETREG1 being ER stress responsive [29] and the ER tubule-localized RETREG3 responsive to nutrient scarcity [37].

### RTN3L

RTN3L (reticulon 3-long) is member of the reticulon family of ER-membrane shaping proteins and is the other known RHD-containing reticulophagy receptor in addition to the RETREG-family proteins [38] (Figure 1). There are four human RTN genes that each encode multiple protein products via alternative pre-mRNA splicing. The long splice isoform of RTN3 (reticulon 3), RTN3L, homodimerizes and induces ER tubule fragmentation in response to starvation stress and recruits nascent autophagosomes via interactions between its LIR-rich amino-terminal domain (Table 1) and LC3B, GABARAP, or GABARAPL1 [38]. This domain can also bind RAB9A, resulting in the recruitment of RTN3L to ER-endosome membrane contact sites during endosome maturation [39]. Here, RTN3L participates in “wrapping” the tubular ER around RAB9A-positive endosomes and regulates the sorting of endosomal cargo like epidermal growth factor receptor and the cation-independent mannose 6-phosphate receptor. These two divergent roles for RTN3L appear to be governed by a stress-dependent switch; in nutrient-rich environments, RTN3L binds RAB9A and controls endosomal maturation and cargo sorting at tubular ER-endosomal membrane contact sites, whereas nutrient depletion redirects RTN3L to ER tubule fragmentation and reticulophagy. RTN3L binding to RAB9A could also aid reticulophagy flux, since RTN3L is incorporated into nascent autophagosomes in a topology that should permit interactions with RAB9A at late endosomes and/or lysosomes.

The first RTN3L substrate proteins were recently discovered through the study of misfolded pro-hormones; RTN3L was required for reticulophagy mediated clearance of aggregation-prone mutant forms of pro-insulin, POMC (pro-opiomelanocortin-alpha), and pro-arginine-vasopressin [40]. However, because RTN3L has only two short ER lumenal loops joining transmembrane domains located within the lumenal side of the ER-lipid bilayer [41,42], it has a limited ability to directly interact with these substrate proteins. Thus, the current model is that RTN3L binding proteins with longer ER-lumenal domains may assist with target protein recruitment, similar to the way that the ER transmembrane chaperone CANX delivers substrate proteins to RETREG1. The first such candidate is the ER-transmembrane protein PGRMC1 (progesterone receptor membrane component 1), which associates with RTN3L and binds mutant pro-opiomelanocortin via a carboxy-terminal lumenal domain, leading to clearance by reticulophagy [43]. Other mutant prohormones were cleared by RTN3L-dependent reticulophagy in a PGRMC1-independent manner, which hints at the existence of alternative cargo receptors that collaborate with RTN3L in reticulophagy.

## Transmembrane reticulophagy receptors lacking a reticulon homology domain

### ATL proteins

**Figure 2.**
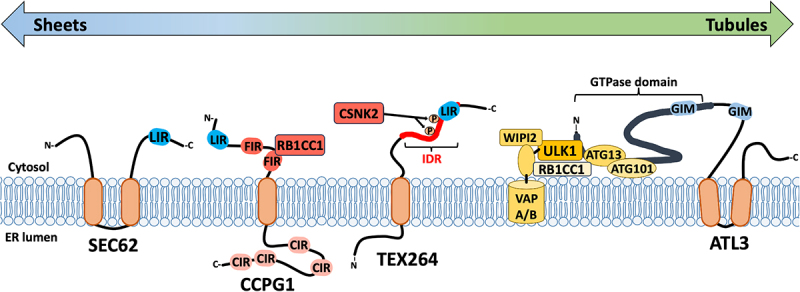
Transmembrane reticulophagy receptors that lack a reticulon homology domain. There are four known transmembrane reticulophagy receptors that lack reticulon homology domains (RHDs); SEC62 and CCPG1 are predominantly found in ER sheets, whereas TEX264 and ATL3 are predominantly found in ER tubules. SEC62 and CCPG1 have cytoplasmic LIRs; CCPG1 also binds RB1CC1 via a FIR domain. TEX264 has an intrinsically disordered region (IDR), highlighted in red; CSNK2-mediated phosphorylation of residues upstream of the LIR in the IDR aids LC3 binding and phagophore recruitment. ATL3 has two GIMs that confer GABARAP binding. ATL3 associates with ATG13 and ULK1 via a GTPase domain (in black). Through its association with ATG13 and ULK1, ATL3 indirectly interacts with VAPA and VAPB, WIPI2, ATG101 and RB1CC1.

In mammals, the atlastin (ATL) family of membrane-bound GTPases is comprised of three members, ATL1, ATL2 and ATL3 (Figure 2). ATLs form homo- and hetero-oligomeric complexes in the tubular ER and regulate ER morphology by connecting ER tubule protrusions to form three-way junctions [44,45]. They can also interact with ER-tubule shaping proteins, such as members of the RTN family. The first clue that ATL3 could function as a reticulophagy receptor was that cellular starvation in ATL3-depleted cells significantly impaired lysosomal degradation of tubular ER proteins such as REEP5 (receptor accessory protein 5) [13]. ATL3 contains two amino-terminal GABARAP-interacting motifs (GIMs) that promote selective binding to GABARAP proteins (GABARAP, GABARAPL1, and GABARAPL2) rather than LC3 proteins, with the highest affinity for GABARAP (Table 1); these GIMs are essential for ATL3-induced reticulophagy [46]. Subsequent studies bolstered the evidence for ATL proteins in reticulophagy by demonstrating that heterodimeric ATL2-ATL3 complexes bind to ULK1 and promote its recruitment to RB1CC1-ATG13-containing puncta on tubular ER membranes, which is followed by ATG101 recruitment and formation of phagophores [47].

### SEC62

In response to ER stress, the UPR drives expansion of the ER compartment to meet protein folding and processing demands [48]. Stress resolution triggers a recovery phase in which ER proteins return to pre-stress levels and excess ER membranes are cleared. In 2016, Fumagalli and colleagues coined the term “recovER-phagy” to describe this process of autophagy-mediated clearance of expanded ER membranes and associated proteins after the resolution of ER stress [49]. They identified SEC62 as a recovER-phagy receptor required for the reestablishment of pre-stress levels of ER proteins like HSPA5/BiP, HSP90B1/Grp94 and protein disulfide isomerases (PDIs). SEC62 is a highly conserved integral ER membrane protein associated with co-translational protein import through the SEC61 translocon [50–52]. Because it is encoded by a housekeeping gene that is unaffected by ER stress, SEC62 is likely diverted from preexisting protein pools to participate in recovER-phagy. It contains a carboxy-terminal LIR that binds lipidated LC3B and initiates micro-autophagy [49,53] (Figure 2; Table 1). This process requires the ATPase CHMP2A/VPS2A that provides the energy required for ESCRT-III-driven membrane remodeling and direct endolysosomal engulfment of SEC62-containing ER-derived vesicles.

### CCPG1

In contrast to the constitutively-expressed reticulophagy receptor SEC62, CCPG1 (cell cycle progression 1) is made in response to ER stress via MIST1, a UPR-responsive transcription factor that is a product of ERN1/IRE1 pathway activation [54–56]. CCPG1 is a type II transmembrane protein that constitutively binds RB1CC1 via two RB1CC1/FIP200 interacting regions (FIRs), FIR1 and FIR2, and localizes to the perinuclear ER [15]. It can also bind GABARAP, LC3B and LC3C via a classical LIR located on the cytosolic amino terminus (Figure 2; Table 1). By contrast, the carboxy-terminal lumenal domain of CCPG1 recognizes ER-lumenal cargo via multiple cargo-interacting regions (CIRs); among these, CIR1 is required for CCPG1-dependent degradation of an artificial ER lumenal aggregation-prone protein, whereas CIR2 is required for CCPG1-dependent degradation of ER-resident protein P3H4 (prolyl 3-hydroxylase family member 4 (non-enzymatic)) [57]. This suggests that once it is upregulated in response to ER stress, CCPG1 recruits multiple reticulophagy substrates concurrently to support recovER-phagy.

### TEX264

TEX264 (testis expressed gene 264 ER-phagy receptor) is a major reticulophagy receptor with a type I transmembrane protein topology and a classical LIR that sits in a carboxy-terminal intrinsically-disordered domain [58] (Figure 2, Table 1). TEX264 accumulates in LC3A/LC3B-positive structures near three-way junctions of ER tubules where it directs basal reticulophagy and is responsible for approximately 50% of total reticulophagy flux during starvation [12]. The interaction between TEX264 and LC3 proteins is regulated by phosphorylation; CSNK2 phosphorylates serine residues just upstream of the TEX264 LIR and enhances interactions with LC3 proteins [59]. TEX264 is also found at the inner nuclear membrane where it associates with DNA-replication forks and participates in the repair of a specific type of nuclear DNA lesion known as a topoisomerase 1 cleavage complex, which is extruded from the nucleus and degraded in lysosomes [60–62].

## Soluble reticulophagy receptors

### CALCOCO1

**Figure 3.**
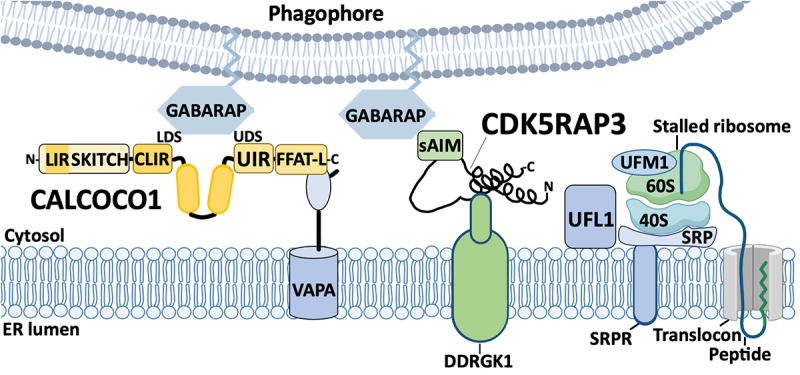
Soluble cytosolic reticulophagy receptors. CALCOCO1 is recruited to the cytosolic face of the ER by binding VAPA/B through its C-terminal FFAT-like domain. CALCOCO1 binds GABARAP via multivalent interactions; its amino-terminal CLIR interacts with the GABARAP LIR-docking site (LDS) and its carboxy-terminal UIR interacts with the GABARAP ubiquitin interacting motif docking site (UDS). CDK5RAP3 is a core component of the UFMylation machinery that directs ribosomal protein UFMylation during stalling at the ER translocon; following ribosomal protein UFMylation, CDK5RAP3-DDRGK1/UFBP1 complexes can be released from this role, and bind GABARAP via a sAIM motif in CDK5RAP3, thereby promoting phagophore recruitment for reticulophagy. SRP, signal recognition particle; SRPR, signal recognition particle receptor.

CALCOCO1 (calcium binding and coiled coil domain 1) is a soluble cytosolic reticulophagy receptor [63] and a paralog of well-known selective autophagy receptors TAX1BP1 (Tax1 (human T cell leukemia virus type I) binding protein 1) and CALCOCO2. CALCOCO1-mediated reticulophagy activity requires formation of homo-oligomers and involves a non-canonical LC3C-interacting region called a “CLIR” [64,65], and a ubiquitin-interacting motif (UIM) that contribute to binding GABARAP and other Atg8-family proteins [63,66] (Figure 3, Table 1). In response to starvation or proteotoxic stress, CALCOCO1 homo-oligomers are recruited to ER tubules by the integral ER membrane proteins VAPA (vesicle-associated membrane protein, associated protein A) and VAPB (vesicle-associated membrane protein, associated protein B and C); this interaction requires a VAP-interacting motif denoted FFAT (phenylalanines (FF) and an acidic tract (AT)) [67,68]. Thus, binding VAPA and VAPB allows the soluble cytosolic CALCOCO1 protein to target tubular ER for degradation.

### CDK5RAP3/C53

Recently, Stephani and colleagues discovered a second cytosolic reticulophagy receptor, CDK5RAP3/C53 (CDK5 regulatory subunit associated protein 3) [69]. CDK5RAP3 was identified as a highly conserved Atg8-interacting protein in *Arabidopsis*, and studies of human CDK5RAP3 confirmed binding to several Atg8-family proteins including GABARAP and GABRAPL1. It features three non-canonical LIRs with shuffled Atg8 Interacting Motifs (sAIMs) located in an intrinsically disordered region (Figure 3, Table 1). CDK5RAP3 localizes to the ER and colocalizes with GABARAP in response to ER stress-inducing stimuli like tunicamycin, but not general macroautophagy stimuli like carbon starvation. Furthermore, treatment with the lysosomal inhibitor Bafilomycin A1 caused enlargement of C53-GABARAP foci at the ER. Together, these observations clearly establish CDK5RAP3 as a *bona fide* reticulophagy receptor.

CDK5RAP3 also participates in a ubiquitin-like protein post-translational modification known as UFMylation that operates at the surface of the ER and is regulated by ER stress [70]. In UFMylation, UFM1 (ubiquitin-fold modifier 1) is conjugated to lysine residues on target proteins by a multi-subunit E3 ligase complex that includes CDK5RAP3. Ribosome stalling during translocation of nascent proteins at the ER is a cue for UFM1 conjugation to ribosomal protein RPL26, which sends stalled nascent chains for lysosomal degradation [70–73]. The direct involvement of CDK5RAP3 in UFMylation provided a natural avenue for investigating physiologic triggers of CDK5RAP3-mediated reticulophagy. Interestingly, in the context of UFM1 E3 ligase complexes on the cytosolic face of the ER, CDK5RAP3 sAIMs bind UFM1 directly, which prevents association with GABARAP proteins. However, when ribosome stalling causes RPL26 UFMylation, the CDK5RAP3 sAIMs are liberated and able to bind GABARAP proteins. This competitive relationship was confirmed in a study wherein the CDK5RAP3 sAIM was substituted with a canonical LIR that increased GABARAP binding and decreased UFM1 binding; this substitution resulted in premature activation of CDK5RAP3-mediated reticulophagy [74]. Together, these studies suggest that RPL26 UFMylation licenses downstream reticulophagy responses.

## Mechanistic links between the UPR and reticulophagy

### Overview of the UPR in the context of reticulophagy

The UPR is coordinated by three transmembrane ER sensors called EIF2AK3/PERK, ERN1/IRE1, and ATF6. Each sensor binds a chaperone called HSPA5/BiP in the ER lumen [75,76]. When the burden of unfolded proteins exceeds ER folding capacity, HSPA5 dissociates from UPR sensors to aid protein folding, resulting in sensor activation and the synthesis of bZIP transcription factors that translocate to the nucleus and orchestrate a transcriptional response that reduces the influx of newly synthesized proteins to the ER, accelerates the degradation of misfolded proteins, and increases the amount ER-resident chaperones and enzymes that assist protein folding and modification. This transcriptional program includes gene products involved in macroautophagy, including reticulophagy receptors. In the following section we will review each arm of the UPR and how they influence ER stress-induced autophagy and/or reticulophagy.

### EIF2AK3

EIF2AK3 is an ER-localized transmembrane UPR sensor with a lumenal amino-terminal domain that binds HSPA5 and a cytosolic carboxy-terminal kinase domain [77]. EIF2AK3 monomers bound to HSPA5 are inactive. When unfolded proteins interact with the substrate binding domain of HSPA5 it dissociates from EIF2AK3, thereby triggering EIF2AK3 dimerization, trans-autophosphorylation, and the formation of higher-order oligomers. This activated form of EIF2AK3 phosphorylates EIF2A/eIF2α, which inhibits cap-dependent protein synthesis at the initiation step by preventing recycling of the EIF2-GTP-tRNA_i_^Met^ ternary complex. Global translation inhibition is a key aspect of the ER stress response that reduces folding burden to give the UPR a better opportunity to increase capacity and restore proteostasis. The reduced availability of ternary complexes alters 40S ribosomal subunit scanning to allow upstream ORF/uORF skipping, a process whereby start codons for small upstream ORFs are bypassed so the 40S ribosomal subunit can access downstream start codons to translate an ORF [78]. In this way, ER stress enables synthesis of proteins like ATF4 (activating transcription factor 4) by upstream ORF skipping. ATF4 is a basic leucine zipper domain transcription factor that transactivates genes that encode proteins involved in amino acid import and protein folding, thereby aiding the restoration of ER proteostasis. ATF4 boosts cellular catabolic activity to reduce the burden of protein folding in the ER by transactivating an array of autophagy-related genes including *MAP1LC3A, MAP1LC3B, MAP1LC3B2, ATG3, ATG5, ATG7, ATG10 ATG13, ATG16L1, BECN1* and *WIPI1* [79–81]. ATF4 also transactivates *CCPG1* [81], implicating reticulophagy as a downstream consequence of EIF2AK3 activation. There is evidence that that the reticulophagy receptor SEC62 stimulates autophagy in an EIF2AK3- and ATF4-dependent manner, although precise mechanisms remain obscure [82].

### ERN1

**Figure 4.**
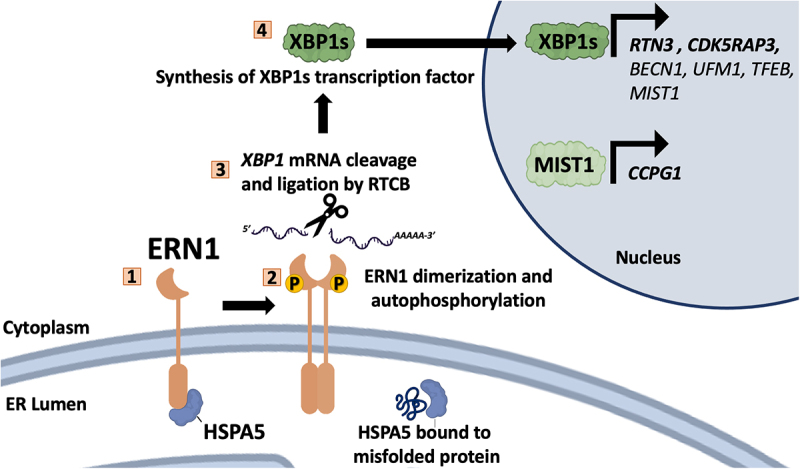
The unfolded protein response (UPR) increases reticulophagy receptor levels. ERN1 is an ER localized transmembrane protein that is inactive when bound to the ER lumenal chaperone HSPA5. HSPA5 dissociates from ERN1 in response to the accumulation of unfolded proteins in the ER lumen, which allows ERN1 activation via dimerization and trans-autophosphorylation. ERN1 is an RNA endonuclease that cleaves cytosolic *XBP1* mRNA at two proximal hairpin loops; cleavage products are ligated by the RCTB tRNA ligase, which removes a 26-base intron, and shifts the reading frame, allowing synthesis of the XBP1s transcription factor with a carboxy-terminal bZIP transactivation domain. XBP1s translocates to the nucleus and transactivates an array of UPR genes, including genes encoding the RTN3 and CDK5RAP3 reticulophagy receptors, and the MIST transcription factor, which in turn transactivates the gene that encodes the CCPG1 reticulophagy receptor.

ERN1 is a serine/threonine kinase and endoribonuclease that exists as an inactive homodimer in resting cells [83–85]. During ER stress, HSPA5 dissociates from ERN1, which triggers ERN1 oligomerization and trans-autophosphorylation [86] (Figure 4). Active ERN1 has sequence-specific endoribonuclease activity that cleaves the cytosolic *XBP1* mRNA at two sites, generating fragments that are subsequently ligated by RTCB, the catalytic subunit of the mammalian tRNA ligase complex, removing an intervening “intron” RNA segment [87]. This cytosolic mRNA splicing event changes the protein products of the mRNA. In unstressed cells, un-spliced *XBP1* (X-box binding protein 1) mRNA encodes XBP1 un-spliced (XBP1u), which has a short half-life and lacks transcriptional activity. By contrast, in response to ER stress, the spliced *XBP1* mRNA encodes XBP1 spliced (XBP1s) protein that has an additional carboxy-terminal transactivation domain and a nuclear localization sequence/NLS. Upon translocation to the cell nucleus, XBP1s transactivates a variety of stress-response genes including those that encode ER chaperones and components of the ER-associated degradation (ERAD) system [88,89], as well as the key autophagy protein BECN1 [90]. Interestingly, after products of ERN1 activation increase *Becn1* transcription, ERN1 may assist BECN1-dependent autophagy through a transcription-independent mechanism [91]. Specifically, active ERN1 recruits TRAF2 (TNF receptor-associated factor 2) and MAP3K5/ASK1 (mitogen-activated protein kinase kinase kinase 5) protein complexes to the ER, where they phosphorylate and activate MAPK/JNK (mitogen-activated protein kinase) [91], which in turn phosphorylates and inactivates BCL2 [92]. MAPK/JNK-dependent phosphorylation and inactivation of BCL2 was previously demonstrated to alleviate the inhibitory effect of BCL2 binding BECN1, allowing for initiation of BECN1-dependent autophagy [93]. Thus, ERN1 activation appears to orchestrate BECN1-dependent autophagy by two mechanisms: (i) generating XBP1s that transactivates *BECN1*, and (ii) initiating a signaling cascade that results in JNK-mediated phosphorylation of BCL2.

In response to ER stress, XBP1s also transactivates *Ufm1* as well as genes encoding the core UFMylation machinery including *Ufm1*, *Uba5*, *Ufl1*, and *Cdk5rap3* [94]. Accumulating evidence indicates that the primary function of protein UFMylation is in regulating ER proteostasis, whereby co-translational UFMylation of the ribosomal protein RPL26 at the ER translocon directs lysosomal degradation of nascent polypeptides by reticulophagy [95].

Several reticulophagy receptors are regulated by ERN1 (Figure 4). The strongest evidence for ERN1 regulation of reticulophagy involves XBP1s-driven production of the BHLHA14/MIST1 transcription factor that transactivates *CCPG1* [54–56]. XBP1s also transactivates *TFEB*, which encodes a transcription factor that in turn transactivates genes involved in lysosome biogenesis and autophagy [96,97]; RETREG1 is also a TFEB target gene [98], although no firm links to ERN1 activity have been established to date. Finally, RTN3L production has been linked to ERN1 activity, which is supported by the observation of XBP1s response elements in the *RTN3* promoter [99].

### ATF6

ATF6 is a monomeric ER-localized type II transmembrane protein; ER stress promotes the formation of disulfide bond-linked ATF6 homodimers that migrate to the Golgi [100], where they are cleaved by MBTPS1/S1P (membrane-bound transcription factor peptidase, site 1) and MBTPS2/S2P, liberating the cytosolic amino-terminal ATF6 fragment known as ATF6-N [101]. ATF6-N is a bZIP transcription factor that transactivates genes with products involved in protein folding and ERAD [102]. ATF6-N also transactivates *Ddit3/Chop* and *Xbp1*, providing a striking example of crossover between the three arms of the UPR, and an avenue for ATF6-mediated control of autophagy through these downstream transcription factors [102,103]. ATF6 has also been shown to play a role in the regulation of autophagy by IFNG/IFN-γ (interferon gamma), whereby hetero-dimers comprised of ATF6-N and the IFNG responsive transcription factor CEBPB/C/EBP-β upregulate DAPK1 (death associated protein kinase 1), which in turn phosphorylates BECN1 and induces autophagy [104]. Despite these clear mechanistic links between the ATF6 ER stress sensor and autophagy, ATF6-mediated control of reticulophagy remains unexplored to date.

In summary, each branch of the UPR promotes autophagy and, in some cases, clear mechanistic links to reticulophagy have been established. Many viral infections cause ER stress [105,106], but our understanding of reticulophagy in host antiviral defenses is only beginning to emerge.

## xERophagy: a new form of immune regulation at the endoplasmic reticulum

Immediately upon infection, viruses are faced with pattern recognition receptors (PRRs) that monitor the intracellular environment for molecular signatures of infection [107]. PRRs can initiate a process called “xenophagy” to support the innate immune response by delivering whole pathogens or pathogen components to lysosomes for degradation [108,109]. Recently, a new virus-specific form of xenophagy was described, which involves the decoration of virus-containing endosomes with sorting nexin protein 5 and recruitment of the ATG14-containing class 3 phosphatidylinositol 3-kinase complex I/PI3KC3-C1 lipid kinase complex required to initiate autophagosome formation [110]. Even though many viruses usurp ER processes and rearrange ER membranes, no analogous form of virus-specific autophagy at the ER has been described to date. Nevertheless, there is accumulating evidence that reticulophagy supports antiviral responses. In the following section, we will discuss how PRRs and xenophagy are coordinated to initiate an antiviral response and collaborate with reticulophagy to protect host cells from viral infections.

### Innate immunity: xErophagy mediated degradation of viral components

One of the earliest documentations of xenophagy was the 1998 discovery that Sindbis virus (SINV) infection is limited by BECN1 in murine brains [111]; the word “autophagy” was not mentioned in this study because BECN1’s role in autophagy was not established until the following year [112]. Many viruses have since been shown to be targeted by xenophagy and molecular mechanisms of virus targeting have been elucidated. For SINV, capsid proteins are bound by the selective autophagy receptor SQSTM1/p62 and targeted to the autophagosome for degradation [113], which is a typical mechanism for the xenophagy of viruses.

Although xenophagy has been documented for nearly 30 years, the study of reticulophagy-dependent xenophagy remains in its infancy. Discoveries of diverse reticulophagy receptors and linked signaling pathways have provided new tools to investigate how reticulophagy affects viral infection. In the following section, we will refer to reticulophagy-dependent xenophagy as “xERophagy” (pronounced “zee-row-phagy”) and discuss how xERophagy contributes to host responses to infection. We propose that xERophagy is a distinct subset of xenophagy that requires a reticulophagy receptor to initiate the degradation of pathogen components or whole pathogens during infection. We will focus on how xERophagy interacts with viruses for the purpose of this article.

RETREG1-mediated xERophagy has been shown to limit the replication of coronavirus (CoV) [16], dengue virus (DENV) [19,20], Ebola virus (EBOV) [18], West Nile virus (WNV) [19], and Zika virus (ZIKV) [19,20,114]. The first documentation of RETREG1-dependent xERophagy involved the discovery of increased EBOV replication in RETREG1-deficient cells [18]. Upon EBOV infection, *retreg1* knockout cells displayed abnormally large inclusions that contained the viral nucleoprotein, which suggested that RETREG1 normally targets ER inclusions enriched in viral components for degradation to limit EBOV replication.

**Figure 5.**
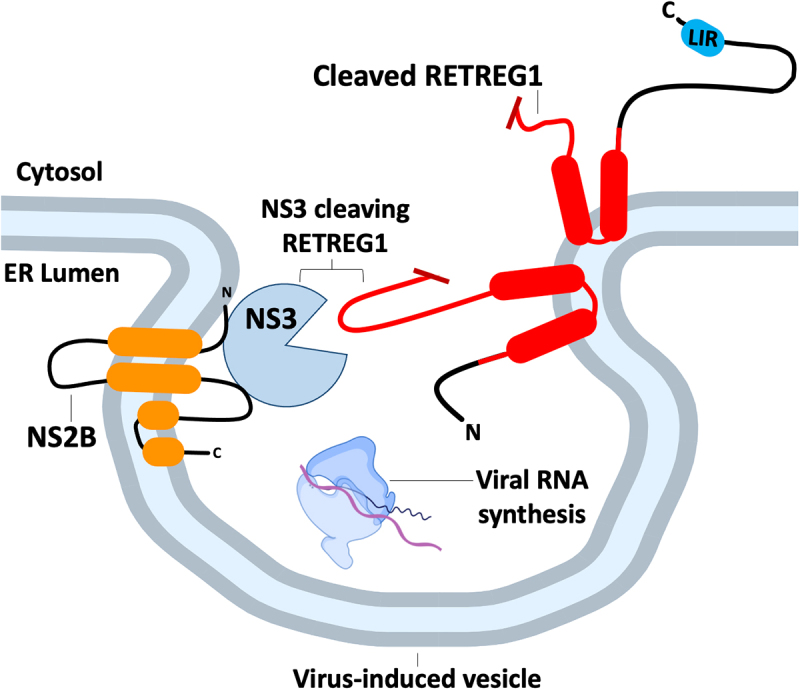
Flavivirus proteases cleave RETREG1 to prevent xERophagy. Several flaviviruses replicate more efficiently in cells deficient in RETREG1. Flavivirus RNA synthesis takes place in arrays of ER invaginations known as “vesicle packets”, where the viral NS3 protease is tethered to the cytosolic face of the ER by the viral transmembrane adapter protein NS2B. NS3 inactivates RETREG1 by cleaving a loop in the RHD (in red). Preventing RETREG1 cleavage by mutating the protease cleavage site restores xERophagy of viral components.

Flaviviruses remodel the ER to create replication compartments that segregate viral genome replication and assembly of progeny virions away from the cytosol [115,116]. These replication compartments: (i) concentrate viral components to facilitate genome replication and particle assembly, and (ii) shield these viral components from intracellular sensors that would otherwise trigger anti-viral responses. Usurpation of the ER to create replication compartments puts flaviviruses in a position to interact with reticulophagy machinery (Figure 5). RNAi-mediated silencing of *retreg1* enhanced DENV and ZIKV replication [19], suggesting that RETREG1 has antiviral properties against flaviviruses. Flaviviruses encode a protease complex consisting of the protease non-structural (NS) 3 (NS3) and co-factor NS2B. This viral protease complex cleaves and disables RETREG1, yielding a carboxy-terminal RETREG1 fragment that can bind LC3 but cannot oligomerize or induce ER membrane curvature. Interestingly, a cleavage-resistant RETREG1 mutant construct colocalized with the viral protease and enhanced reticulophagy. These findings clearly demonstrate that RETREG1 participates in xERophagy, which is supported by studies of BPIFB3 (BPI fold containing family B, member 3), an ER-resident autophagy regulator that suppresses RETREG1-dependent reticulophagy, thereby facilitating DENV and ZIKV replication [20].

xERophagy restricts CoV infection by limiting the formation of ER-derived double membrane vesicles (DMVs) that serve as viral replication organelles [16]. This is countered by ORF8 proteins (encoded by both SARS-CoV-1 and SARS-CoV-2) that bind to SQSTM1 and direct sequestration of reticulophagy receptors like RETREG1 and ATL3 in protein condensates. This situation is further complicated by the CoV ORF3a protein that localizes to multiple compartments in the endomembrane system, including the ER, and plays an important role in egress of newly assembled CoV progeny viruses. ORF3a triggers ER stress and recruits HMGB1 (high mobility group box 1) to bind BECN1 and initiate the synthesis of autophagic membranes for viral egress [17,117]. Thus, ORF8-mediated suppression of RETREG1-dependent xERophagy may be especially important for CoV replication in the context of concomitant expression of autophagy-inducing viral protein like ORF3a.

Hepatitis C virus (HCV) is another flavivirus that has also been shown to interact with reticulophagy machinery. Years before identification of RTN3 as a reticulophagy receptor, it was shown to negatively regulate hepatitis C virus (HCV) replication [118]. RTN3 binds the viral non-structural protein NS4B [119], which is required for the formation of membranous webs made up of DMVs that are sites of viral polyprotein processing and genome replication [120–123]. RTN3 binds the second amphipathic α-helix/AH2 of NS4B, and thereby inhibits the NS4B homo-oligomerization required for membranous web formation [124]. Viral mechanisms to antagonize this function of RTN3 remain to be fully elucidated but may involve another amphipathic α-helix in NS4B called A1 that appears to limit RTN3-NS4B binding [118].

### Innate immunity: reticulophagy and pattern recognition receptors

At the cellular level, PRRs coordinate a complex first line of defense against invading viruses and bacteria. PRRs recognize pathogen associated molecular patterns (PAMPs) and initiate signal transduction events that elicit the synthesis of antiviral factors [107]. There is accumulating evidence that the ER serves as a platform for coordination for PRR-driven antiviral responses. Chief among these is the CGAS (cyclic GMP-AMP synthase-STING1 pathway (reviewed in [125]). CGAS is a PRR that patrols the cytoplasm; binding of viral double-stranded DNA (dsDNA) activates CGAS enzymatic activity, generating the second messenger cyclic GMP-AMP (cGAMP) [126,127]. STING1 is a dimeric ER resident protein with a large cytosolic ligand-binding domain; binding of cGAMP closes the ligand-binding domain and triggers a dramatic conformational change in STING1 whereby the ligand-binding domain rotates 180° with respect to its transmembrane domain, allowing the formation of tetramers and higher-order oligomers that exit the ER [128,129]. In the *trans* Golgi, these STING1 oligomers are phosphorylated by TBK1 (TANK-binding kinase 1); TBK1 also phosphorylates and activates the IRF3 (interferon regulatory factor 3) transduction and along with IKBKE/IKK-epsilon initiates NFKB signal transduction required to support strong antiviral gene expression. Interestingly, the control of viruses by the CGAS-STING1 pathway is not limited to those with dsDNA genomes, as certain RNA viruses cause damage to host cell nuclei or mitochondria, resulting in the release of host dsDNA into the cytoplasm to serve as PAMPs for CGAS [130–132].

**Figure 6.**
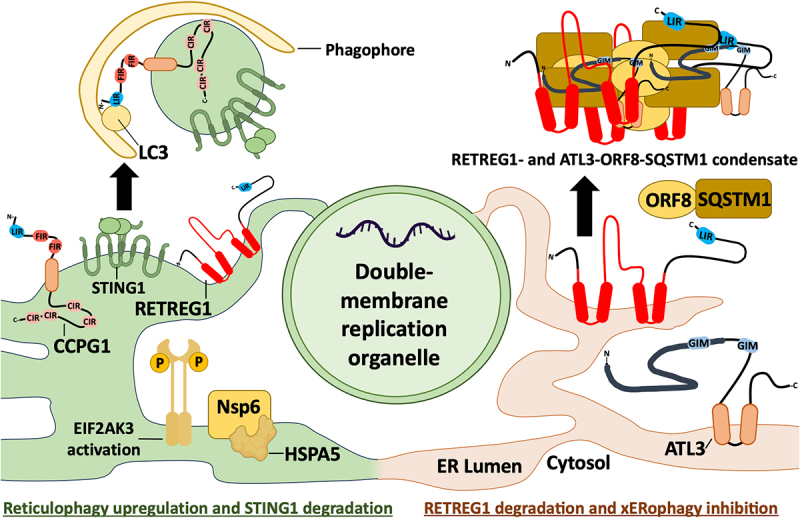
Coronaviruses co-opt reticulophagy receptors for STING1 degradation and then sequester them in protein condensates to prevent xERophagy. In the early stages of coronavirus infection, NSP6 assists in formation of the double-membrane replication organelle, where viral RNA synthesis takes place. NSP6 also assists viral replication by activating EIF2AK3-dependent reticulophagy, involving the RETREG1 and CCPG1 receptors that target STING1 for lysosomal degradation. However, excessive reticulophagy limits replication organelle formation. This is countered by the viral protein ORF8, which binds to RETREG1 or ATL3, along with the selective autophagy receptor SQSTM1, to sequester these reticulophagy receptors in insoluble condensates, thereby inhibiting xERophagy.

Considering the important role that the CGAS-STING1 pathway plays in orchestrating antiviral responses, it should come as no surprise that viruses have evolved mechanisms to limit pathway activation, and the position of STING1 in ER membranes makes it a prime candidate for control by reticulophagy. For example, SARS-CoV-2 NSP6 is a transmembrane glycoprotein involved in sculpting the ER into replication organelles (Figure 6). NSP6 activates EIF2AK3 and triggers RETREG1- and CCPG1-dependent reticulophagy, which targets STING1 for lysosomal degradation [133]. In this way, NSP6 harnesses reticulophagy to suppress CGAS-STING1-mediated antiviral responses, at least in the early stages of infection until sufficient ORF8 accumulates to consign RETREG1 to protein condensates [16]. By contrast, the picornaviruses foot-and-mouth disease virus (FMDV) and Seneca valley virus (SVV) induce UPR-dependent autophagy to support viral replication [134–136], but ultimately also consign STING1 to lysosomal degradation via RETREG1-dependent reticulophagy [137].

Altogether, these studies provide compelling evidence for viral restriction by xERophagy and countermeasures deployed by viruses to prevent xERophagy. In the next section, we will review the evidence for how viruses can usurp reticulophagy machinery to enhance viral fitness.

## The viruses strike back: pro-viral reticulophagy interactions

### Home sweet home: viruses hijack reticulophagy receptors to build replication compartments

Many RNA viruses create ER-membrane-derived replication compartments where viral RNA synthesis takes place in an optimized environment, with ample viral enzymes, nucleic acid templates, and ribonucleotides in place. This work is conducted in secret, with viral RNA products hidden away from PRRs. This phenomenon was first observed in poliovirus infection models with the accumulation of DMVs that were subsequently shown to be sites of viral RNA encapsidation [138,139]. Years later, study of the minimal viral determinants of DMV formation shed new light on the links between DMVs and autophagy; ectopic expression of key poliovirus proteins was sufficient to create DMVs, and these structures contained LC3 and LAMP1 (lysosomal-associated membrane protein 1), suggesting an autophagosome or lysosome origin [140–142]. The emerging model is that many viruses usurp autophagy machinery in a similar fashion to create replication compartments, but the contribution of reticulophagy receptors remains understudied to date, in part because many of these receptors have only recently been discovered. Such studies will inform our understanding of how viruses usurp ER membranes and autophagic machinery while avoiding the fate of lysosomal degradation.

The atlastin proteins, including the reticulophagy receptor ATL3, are ER-shaping GTPases that play roles in supporting the replication of diverse viruses. For flaviviruses like DENV and ZIKV, ATL3 is recruited to nascent sites of viral replication to enhance the formation of ER-derived compartments [143–145]. ATL3 may also support flavivirus particle maturation by increasing retrieval of furin proteases from the plasma membrane [146], although this requires further confirmatory evidence. Furthermore, it remains to be seen whether ATL3’s role as a reticulophagy receptor impacts flavivirus replication.

**Figure 7.**
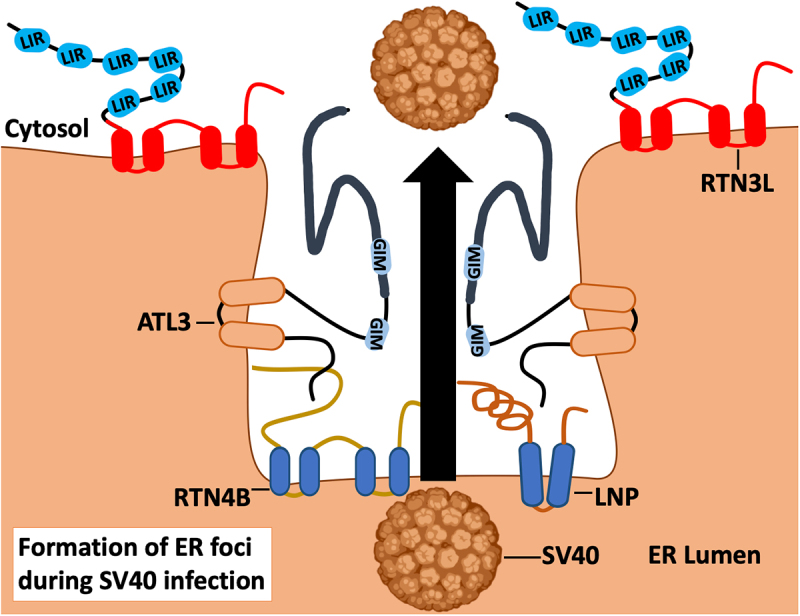
ATL3 and RTN3L support the formation of SV40 foci during ER escape. SV40 polyomavirus enters cells via a CAV-dependent pathway that delivers virus capsids to the ER lumen. To gain access to the cytosol, SV40 penetrates the ER membrane at foci comprised of multi-tubular ER junctions. Formation of these junctions requires the GTPase activity of ATL3, which assists membrane fusion. ATL3 associates with SV40 VP1 protein, as well as host proteins LNP and RTN4B, to yield a membrane-penetration complex at these sites. RTN3L stabilizes these ER foci and assists membrane penetration by remodeling ER membranes to relieve mechanical stress.

A member of the *Polyomaviridae* family of viruses called simian virus 40 (SV40) also requires ATL3 ER-shaping activity to remodel the ER during infection (Figure 7). SV40 enters cells through CAV (caveolin)-dependent endocytosis and accesses the ER lumen, where the viral particle is destabilized and then penetrates the ER membrane [147–155]. This penetration step occurs at ER membrane “foci”, which consist of multi-tubular ER junctions supported by LNP (lunapark, ER junction formation factor), a host protein that normally assists in the formation of three-way junctions in the tubular ER [156–158]. ATL3 plays an important role in this process by forming a complex with LNP and RTN4 and associating with the SV40 capsid protein VP1 to form a membrane-penetration complex [159]. In addition to ATL3, RTN3 is recruited to foci during SV40 infection to facilitate ER-membrane curvature and protect ER-membrane integrity upon SV40 exiting the ER-membrane [160]. RTN3 activity also relieves mechanical stress incurred on the ER-membrane during ER-to-cytosol membrane penetration. Thus, RTN3 is required to maintain ER-membrane integrity and stabilize foci to combat the mechanical stress imposed by the relatively large SV40 capsid being pulled through small transient pores in the ER membrane. It remains to be determined how SV40 capsids co-opt reticulophagy receptors for penetration while evading reticulophagy-mediated lysosomal degradation.

Reticulon-family proteins also support viral replication compartment formation, with RTN3L used by picornaviruses [161] and flaviviruses [162], and RTN3 or RTN4 used by CoVs [163]. 2C is an essential conserved picornavirus protein with RNA binding activity that binds to RTN3L at ER membranes to support DMV formation [161]. By contrast, the flaviviruses WNV, DENV, and ZIKV require RTN3.1A (full-length RTN3) to remodel ER-membranes to create viral replication compartments [162]. RTN3.1A depletion was deleterious for flavivirus replication, but affected outcomes differently, with WNV infected cells displaying reduced ER membrane alterations and viral replication compartments, and DENV infected cells displaying elongated replication compartments and an increased number of immature viral particles. RTN3.1A depletion had the most dramatic effect on ZIKV, with a complete loss of replication complexes. RTNs play a similar supportive role in CoV replication. CoV ER-membrane remodeling proteins NSP3, NSP4 and NSP6 are required for replication organelle formation [164–167] and recruit RTN3 or RTN4 to aid in this activity [163]. Structure/function studies indicate that the RHD-containing carboxy-terminal domain of RTN3 or RTN4 is sufficient to support replication organelle formation, whereas the amino-terminal LIRs are dispensable. Thus, for both CoVs and picornaviruses, reticulon-family proteins play essential supportive roles in viral replication, although potential roles for reticulophagy in these processes remain to be investigated.

In contrast to the supportive role that RTN-family reticulophagy receptors play in CoV and picornavirus infection, the biogenesis of DENV replication compartments requires the inhibition of reticulophagy [22]. This role for reticulophagy was revealed by the study of UBE2G2, an E2 ubiquitin conjugating enzyme that supports flavivirus infections due to its role in virus-induced autophagy of lipid droplets (referred to as lipophagy) which is required for the assembly and secretion of viral progeny [168,169]. Replication compartments were absent in Ube2g2 depleted cells infected with ZIKV and instead autophagosome-like double membrane vesicles and lysosomes accumulated [22]. A survey of reticulophagy receptors in UBE2G2-depleted cells revealed that RTN3, RETREG1 and RETREG3 were unaffected, whereas ATL3 levels were decreased and SEC62 levels increased, triggering SEC62-dependent reticulophagy. Importantly, SEC62 silencing rescued virus production in UBE2G2-depleted cells, indicating that SEC62-mediated reticulophagy inhibits viral replication, likely by interfering with viral replication compartments.

### Being a virus is stressful!

Like all gammaherpesviruses, Kaposi’s sarcoma-associated herpesvirus (KSHV) can establish a long-term persistent infection in lymphocytes through latency, a semi-quiescent infectious state in which the viral genome persists as a nuclear episome supported by a modest latent gene expression program [170,171]. KSHV has an elegant mechanism to reactivate from latency in response to ER stress. Specifically, XBP1s transactivates *RTA*, the gene encoding the KSHV lytic switch protein replication and transcription activator, which in turn transactivates KSHV early genes to kick off productive lytic replication [172–174]. A similar dynamic exists for another gammaherpesvirus, Epstein-Barr virus, which also encodes XBP1s-responsive genes that support lytic replication [175]. There is emerging evidence that this ER stress-driven latent/lytic switch mechanism can be regulated by atlastin proteins. Specifically, ectopic expression of ATL1, ATL2, or ATL3 triggered KSHV reactivation from latency, whereas ATL gene silencing had the opposite effect, suppressing stress-induced lytic reactivation [176]. While these studies do not directly implicate reticulophagy in the herpesviral latent/lytic switch mechanism, the discovery of roles for ATL-family proteins provides motivation for further research.

### Don’t eat me: viruses hijack reticulophagy

The EBOV structural glycoprotein GP is required for viral attachment and penetration into host cells (reviewed in [177]). In infected cells, the CANX-calreticulin cycle ensures proper folding and processing of GP in the ER, enabling subsequent trafficking in the secretory pathway to sites of virus assembly [17]. However, high levels of GP are cytotoxic, and this same CANX-calreticulin cycle ensures that excess GP is targeted for degradation via a hybrid ERAD/reticulophagy mechanism [17,178]. Specifically, ER mannosidase activity triggers misfolding of excess GP, which directs K27 ubiquitination of the cytoplasmic tail by the ERAD-associated E3 ubiquitin ligase RNF185 and VCP/p97 ATPase-dependent retrotranslocation into the cytoplasm. However, instead of being degraded in the proteasome like other ERAD substrates, GP is recruited to nascent autophagosomes, resulting in its degradation in autolysosomes. The reticulophagy receptor involved in GP degradation has not been identified, but the participation of CANX in this process suggests that RETREG1 could play a role. In this way, excess GP is degraded in a hybrid pathway initiated by ERAD components and completed by reticulophagy components.

### Going extracellular: autophagy mediated viral spread

Exocytosis involves cellular contents exiting the host cell through the fusion of intracellular vesicles with the plasma membrane. Exocytosis is important for the clearance of intracellular contents and the secretion of signaling molecules. Pathways for exocytosis include secretory autophagy, whereby an autophagosome fuses with the plasma membrane [179,180]. Secretory autophagy can be exploited during viral egress to release progeny virions from an infected cell. Viral egress involving secretory autophagy is well-documented for herpesviruses [181,182], picornaviruses [183–186], and paramyxoviruses [187]. In this section, we will review how viruses interact with autophagy during egress and discuss recent work that implicates reticulophagy in this process.

Herpesvirus egress involves two envelope acquisition events (reviewed in [188]). The first envelope originates from the inner nuclear membrane when the virus to buds into the perinuclear space and is lost as the virus buds into the cytosol. The second envelope is acquired in the cytosol, and evidence suggests the involvement of the trans-Golgi network (envelopment at the trans-Golgi network reviewed in [189]). Interestingly, Epstein-Barr Virus (EBV) envelopes contain LC3-II, suggesting interaction with autophagic membranes during egress [181]. Cells depleted of ATG12 or ATG16L1 released lower quantities of EBV particles and viral DNA became “trapped” in the cytosol. There is also evidence that varicella zoster virus (VZV) utilizes autophagy during egress [182]. Unlike EBV, no double membrane envelopes were seen surrounding VZV particles, but rather a single membrane that had characteristics of an “amphisome” (autophagosome + multivesicular body) surrounded a single viral envelope.

Picornaviruses initiate cell-to-cell spread by hijacking extracellular vesicles (EVs), which are normally released by cells to secrete signaling molecules and maintain proteostasis [190,191]. EVs may shield virions from antibodies in extracellular spaces [192] and allow for the delivery of multiple virions to a target cell at one time [193,194]. EV release does not usually kill host cells, unlike the classic model for non-enveloped virus spread, which involves host cell lysis. By overcoming the need for lysis, EVs prevent the secretion of extracellular PAMPs, reducing the chance the virus is detected by the immune system. Encephalomyocarditis virus (EMCV) initiates the release of EVs that contain LC3 [183] and EMCV exploits secretory autophagy to release these virion-containing EVs [184]. The viral leader protein from EMCV stimulates autophagic flux and is required for the formation and release of EVs. Virus-containing EVs secreted during coxsackievirus B3 (CVB3) infection also exploit secretory autophagy [185]. During infection, extracellular “microvesicles” (EMVs) are released that contain viral particles, LC3, and exosome marker FLOT1 (flotillin 1), suggesting they originated from secretory autophagy. Polioviruses also hijack secretory autophagy machinery; LC3 depletion reduces viral spread, whereas autophagy upregulation has the opposite effect [186]. These findings demonstrate that poliovirus cell-to-cell spread relies on secretory autophagy machinery, providing an attractive alternative to transmission by cell lysis.

Paramyxoviruses induce the formation of syncytia (reviewed in [195]). The attachment of measles virus (MeV) and canine distemper virus (CDV) to host cells induces autophagy, but autophagosome-lysosome fusion is inhibited [187]. ATG7 depletion reduced the formation of syncytia, suggesting that autophagy contributes to efficient cell-to-cell spread. This shows that MeV and CDV trigger autophagy upon infection and that autophagy promotes syncytia.

It is now clear that autophagy is involved in viral egress through viral envelope acquisition (EBV, VZV), the release of virus containing EVs (EMCV, CVB3, PV) and the formation of syncytia (MeV, CDV). Yet, regarding the release of EVs, a new puzzle has emerged: How are viruses and viral components selected and loaded into EVs, and what receptors are involved in loading this cargo? Recent work suggests reticulophagy receptors could be involved in loading EVs.

HCV can be transmitted via the release of EVs containing viral genomes [196,197]. Upon HCV EV-mediated egress, RTN3L mediates the loading of viral genomes into exosomes [198]. HCV infected cells exhibit increased accumulation of RTN3L and the short isoform of RTN3, called RTN3S; exosomes released from these cells are enriched for RTN3L. Exosomes isolated from patients infected with HCV are also enriched in RTN3L and RTN3S. RTN3L and RTN3S form a complex with viral dsRNA and the viral protein NS3. The C-terminus of RTN3, found in both RTN3L and RTN3S, mediates the loading of viral RNA into exosomes. Thus, the accumulating evidence indicates that RTN3 isoforms bind replication-competent viral RNA and direct it to exosomes for packaging and release. Moreover, RTN3L being present in exosomes from cell culture and patient serum suggests the protein is retained within exosomes after viral genomes are loaded. Although this process appears reminiscent of RTN3-mediated reticulophagy, the amino-terminal LIRs are dispensable. However, HCV may induce autophagy during the synthesis of DMVs [122]. Perhaps RTN3 is upregulated as the result of autophagy induction and recruits viral genomes as cargo to be sorted to a virus-induced EV reminiscent of an autophagosome.

## Concluding remarks

There is accumulating evidence that reticulophagy plays key roles in host interactions with diverse viruses. We now know that 1) reticulophagy is involved in innate immunity through xERophagy and PRR signaling, and 2) viruses exploit reticulophagy receptors to subvert anti-viral responses, build viral replication compartments and exit cells during viral egress. Viruses are excellent teachers, and our studies of viral interactions with reticulophagy receptors will likely deepen our understanding of their fundamental biology. Several outstanding questions remain to be addressed: why do reticulophagy receptors involved in the formation of viral replication compartments not engage in xERophagy? Do viruses selectively usurp minor reticulophagy receptors like RTN3L to maintain a low profile? When viruses interact with reticulophagy receptors, do they divert them from their primary roles in reticulophagy? Addressing these questions may reveal opportunities to weaponize reticulophagy receptors against these viruses that depend on them.
